# Metagenomic Analysis of Wild Apple (*Malus sieversii*) Trees from Natural Habitats of Kazakhstan

**DOI:** 10.3390/plants14101511

**Published:** 2025-05-18

**Authors:** Aruzhan Mendybayeva, Alibek Makhambetov, Kirill Yanin, Aisha Taskuzhina, Marina Khusnitdinova, Dilyara Gritsenko

**Affiliations:** 1 Laboratory of Molecular Biology, Institute of Plant Biology and Biotechnology, Almaty 050040, Kazakhstan; aruka0302@gmail.com (A.M.); alibekmahambetov@gmail.com (A.M.); yanin.kirill97@gmail.com (K.Y.); ataskuzina@gmail.com (A.T.); germironame@gmail.com (M.K.); 2 Research Center AgriBioTech, Almaty 050040, Kazakhstan; 3 Department of Molecular Biology and Genetics, Al-Farabi Kazakh National University, Almaty 050040, Kazakhstan

**Keywords:** malus sieversii, metagenomic analysis, bacteria, microbiome

## Abstract

Kazakhstan’s rich biodiversity includes diverse apple populations, notably the wild apple tree (*Malus sieversii*) prized for traits like disease resistance and adaptability, potentially aiding breeding programs. Analyzing their microbiomes offers insights into bacterial diversity and how it influences apple tree development, making it a reliable method for understanding ecological interactions. In this research, 334 apple tree samples were collected from different mountain ranges in southeastern Kazakhstan. An analysis using nanopore-based 16S rRNA sequencing showed a distinct similarity in the microbiome compositions of samples from the Zhongar and Ile Alatau mountain ranges, with a predominance of *Pseudomonadaceae*, *Enterobacteriaceae*, and *Microbacteriaceae*. In contrast, samples from Ketmen ridge showed a higher prevalence of *Enterobacteriaceae*. Alongside the less represented *Pseudomonadaceae* family, in the Ketmen ridge region, bacteria of the *Xanthomonadaceae*, *Alcaligenaceae*, and *Brucellaceae* families were also present. Across all regions, beneficial plant-associated bacteria were identified, such as *Pseudomonas veronii*, *Stenotrophomonas geniculata*, and *Kocuria rhizophila*, potentially enhancing plant resilience. However, opportunistic phytopathogens were also detected, including *Pseudomonas viridiflava* and *Serratia marcescens*, particularly in the Ile Alatau region. These findings highlight the complex microbial interactions in *M. sieversii*, thus offering key insights into host—microbe relationships that can inform apple breeding and ecological preservation efforts.

## 1. Introduction

The *Malus sieversii* apple tree is of major socioeconomic importance in Kazakhstan. *M. sieversii* (Ledeb.) M. Roem is originated from Kazakhstan and was discovered by Johan Sievers in the late 18 century. Then, it was first described in 1830 by German-Russian botanist Carl F. von Ledebour [1]. Additionally, Soviet botanist and geneticist Nikolai Vavilov made a great contribution to studying *M. sieversii* during his expeditions in 1920s and 1930s. He identified Kazakhstan as one of the world’s key centers of origin for cultivated plants, emphasizing the region’s genetic richness. Later, Kazakh scientist Aimak Dzhangaliev carried on Vavilov’s legacy through decades of meticulous fieldwork and research. His work in cataloging phenotypic diversity of wild apple of Kazakhstan and researching resistance to diseases provided deeper insights into the species evolutionary significance. Dzhangaliev collaborated with international scientists, including American pomologists, to preserve and utilize *M. sieversii* germplasm in breeding programs worldwide, including U.S. Dept. of Agriculture (USDA) National Plant Germplasm System in 1980s, which created kazakh collection of *M. sieversii* germplasm seedlings from wild apple forests in the 1990s by USDA scientists [2].

Today, *M. sieversii* is considered the main progenitor of the most contemporary cultivated apple varieties, and it plays a key role in breeding programs due to its beneficial traits and high adaptive potential to environmental conditions [2,3]. The primary genetic reserves of the wild apple tree (*M. sieversii*) in Kazakhstan are located in the Tian Shan, Dzhungarian Alatau, and Altai mountain systems [1]. The seeds of wild apples, originating in Central Asia, were disseminated via endozoochory by mounts used by Silk Road traders. The Old Silk Road path ran through West Asia and Mediterranean and connected regions from the Black Sea to western China. It has significantly influenced the evolutionary trajectory of the domesticated apple. In eastern regions, gene flow may have occurred through hybridization with *Malus prunifolia*, *M. baccata*, and *M. sieboldii*, whereas in the west, introgression likely involved *M. turkmenorum* and *M. sylvestris* [4,5]. Currently, *M. sieversii* is considered a threatened species with “vulnerable” conservation status in the Red Book of Kazakhstan [6] and The International Union for Conservation of Nature’s (IUCN) Red List, in which its population trend is reported as decreasing [7]. A decline of wild apple tree’s natural habitat of up to 70% has been reported [8]. The most significant factors contributing to the decline in wild tree populations include habitat loss driven by urban expansion and deforestation, climate change, and increasing pressure from diseases caused by pathogenic bacteria [9]. Among the pathogenic bacteria of apple trees, *Erwinia amylovora* poses great threat to the wild apple forests. It causes a contagious disease that affects trees from the family Rosaceae and induces blossom, spur and shoot blight and wilt, along with fruit rotting. Nevertheless, some studies displayed that *M. sieversii* has a natural occurrent resistance to the pathogen [10]. Both pathogenic and beneficial bacterial communities play a pivotal role in modulating individual plant development and maintaining overall population health by influencing key processes such as growth, germination, stress tolerance, and environmental adaptability. This complex relationship between plants and microbes is present at different levels, such as the rhizosphere, phyllosphere, and endosphere, in which these interactions help plants grow stronger and adapt better to their surroundings. Although there has been substantial progress in *M. sieversii* rhizosphere research [11,12,13,14,15], the phyllosphere and endosphere remain underexplored and require further investigation. This particularly applies to the endophytic microflora, since, compared to the epiphytes, it is usually less affected by outside contaminants and maintains the closest communication with the host organism [16]. The species composition of the microbiota depends on many different factors such as: plant age, developmental stage, environmental factors, plant genotype and variety.

Currently, microbiome studies mostly rely on DNA sequencing methods. Among these methods, nanopore-based technology enables a high-throughput and cost-efficient analysis of metagenomes [17]. Nanopore sequencing is already widely utilized for microbiome analysis in wheat [18], tomato [19], and other plant-related studies [20,21]. In the past five years, over 1000 plant-associated studies utilizing Oxford Nanopore Technology (ONT) for sequencing have been published in the NCBI PubMed database (https://www.ncbi.nlm.nih.gov/ (accessed on 3 March 2025)). Pecman et al., (2022) showed that nanopore sequencing with a MinION device provides data on virus and viroid detection that are comparable to those obtained with the widely used Illumina sequencing method, when suitable library preparation and sequencing protocols are used [22]. Additionally, several advantages of nanopore sequencing were highlighted, including its high performance, real-time data abilities, relatively low sequencing costs per sample, simple library preparation, and shorter turnaround time. Thus, the determination of the microbiome of the apple tree *M. sieversii* will contribute to a general representation of microbial community-host interactions and their significance in terms of the holobiont concept, which views a host organism and all of its associated microorganisms as a single, integrated unit. Hence, the aim of this study was to determine the species profile of the bacterial microbiome of the wild apple tree *M. sieversii*, which is of great cultural, ecological, and economic importance, in different regions of south-eastern Kazakhstan.

## 2. Results

A total of 334 samples were collected from three regions of south-eastern Kazakhstan: Ile Alatau (1300 m and 1585 m a.s.l.), Zhongar Alatau (1255 m and 1400 m a.s.l.), and Ketmen Ridge (1734 m and 1845 m a.s.l.) (Table 1).

**Figure 1:**
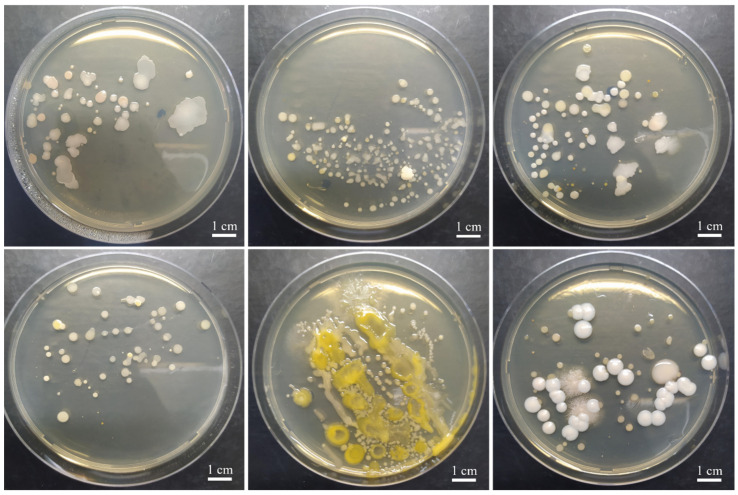
The most abundant bacterial colonies observed across the six populations. The dominant colonies exhibited a round shape and were white or beige in color, while yellow colonies appeared infrequently. Colony sizes ranged from 0.5 to 3 mm in diameter.

The cultivated bacterial extract demonstrated a variety of morphologically different colonies (Figure 1). Most of the isolated colonies were round, opaque, and glistening, with prevailing yellow and gray colors.

After the removal of low-quality reads, the sequencing of samples collected in the foothills of the Zhongar Alatau mountain range yielded 18.5 k total reads, 100% of which were classified with a mean PHRED score of 12.2 (Table 2). Similarly, 71,859 reads from Ile-Alatau and 58,325 reads from Ketmen ridge with identical read quality were obtained. An overview of the processed sequencing data is provided in Supplementary Table S1.

**Figure 2:**
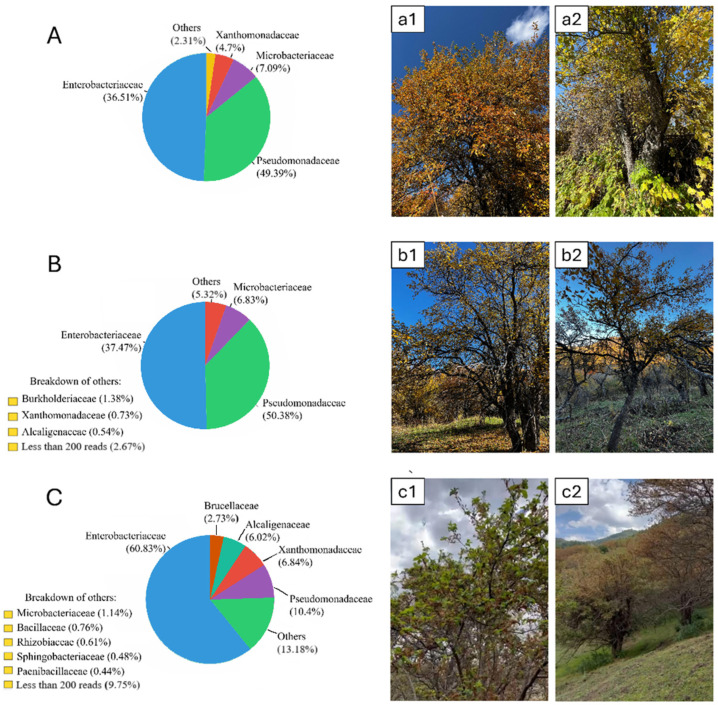
Bacterial microbiomes, expressed as percentages, in different regions. (**A**)—Zhongar Alatau: (**a1**)—phenological site; (**a2**)—genetic reserve of Siever’s wild apple trees. (**B**)—Ile Alatau: (**b1**)—Tau-Turgen; (**b2**)—genetic reserve of Siever’s wild apple trees. (**C**)—Ketmen ridge: (**c1**)—Sumbe; (**c2**)—Ketpentau.

In Zhongar Alatau, the *Pseudomonadaceae* family prevailed with 49.39% presence. The second most abundant family was *Enterobacteriaceae* (36.51%). Additionally, *Xanthomonadaceae* and *Microbacteriaceae* had 4.7% and 7.1% presence, respectively. The Ile Alatau region showcased similar microbial screening results with minor differences in percentages. However, the *Xanthomonadaceae* (0.73%) and *Microbacteriaceae* (6.83%) families were less dominant compared to the *Enterobacteriaceae* and *Pseudomonadaceae* families, which prevailed at 37.47% and 50.38%, respectively. The *Burkholderiaceae* (1.38%) and *Alcaligenaceae* (0.54%) families were also present at relatively small percentages. In contrast, Ketmen ridge showed an increased prevalence of *Enterobacteriaceae* (60.83%). Furthermore, the *Pseudomonadaceae* (10.4%) family appearance ratio was significantly decreased, and the *Xanthomonadaceae* (6.84%) and *Microbacteriaceae* (1.14%) family ratios also changed. Furthermore, the *Alcaligenaceae* (6.02%) and *Brucellaceae* (2,27%) families were represented more significantly compared to the other regions. It is also worth mentioning that families *Bacillaceae* (0.76%), *Rhizobiaceae* (0.61%), *Sphingobacteriaceae* (0.48%), and *Paenibacillaceae* (0.44%) were present (Figure 2).

**Figure 3:**
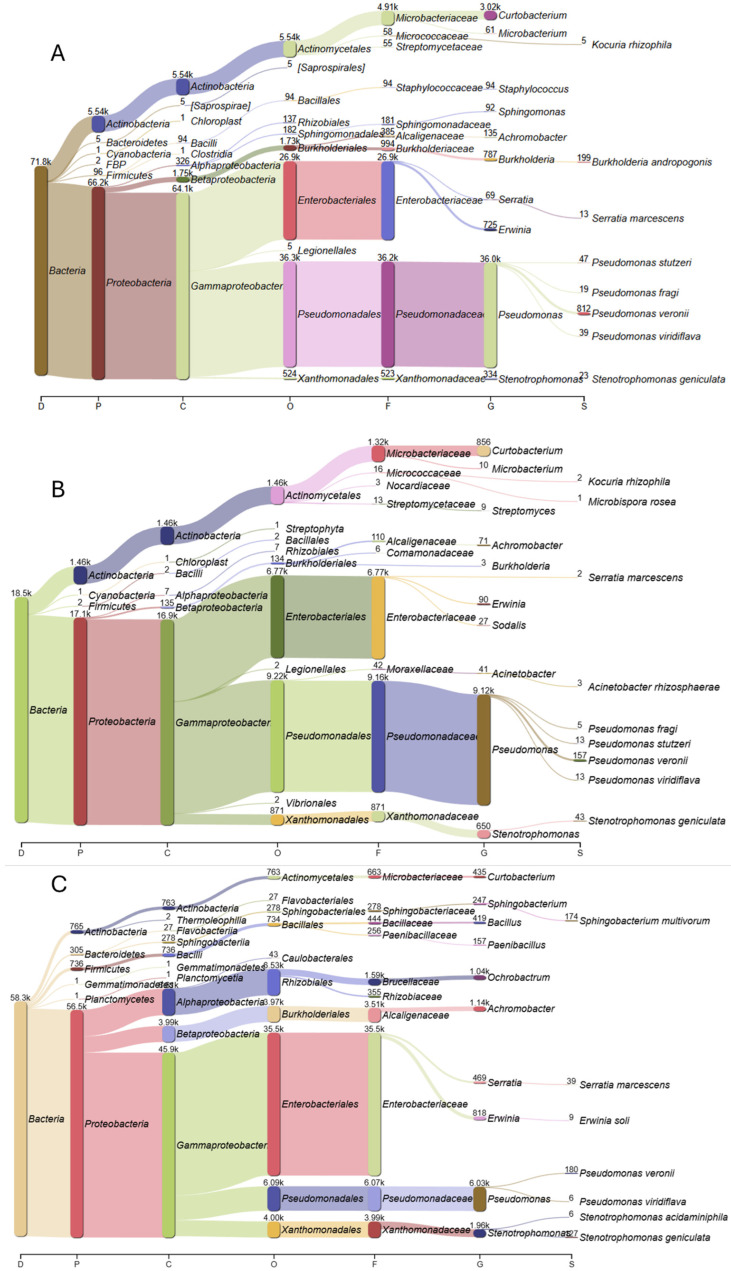
Data discrepancies. Results from sequencing of three regions’ populations. (**A**) Ile Alatau region. (**B**) Zhongar Alatau region. (**C**) Ketpentau region. The colors denote distinct taxonomic groups.

The key species representing the families are shown in Figure 3. For Zhongar Alatau, the most mapped genus from the family *Pseudomonadaceae* was *Pseudomonas* with 9120 reads identified. The most mapped species were *Pseudomonas veronii*, *Pseudomonas viridiflava*, *Pseudomonas stutzeri*, *Pseudomonas fragi*, and *Pseudomonas rhizosphaerae*. The second most mapped genus was *Curtobacterium* (856 reads) from the *Microbacteriaceae* family, followed by *Stenotrophomonas* (650 reads) from *Xanthomonadaceae*, which was represented solely by the *Stenotrophomonas geniculata* species. The highly present *Enterobacteriaceae* family was mapped only to the *Erwinia* and *Sodalis* genera and *Serratia marcescens* species.

For Ile Alatau, *Pseudomonas* was also the most mapped genus, with the same species being identified—*Pseudomonas veronii*, *Pseudomonas viridiflava*, *Pseudomonas stutzeri*, *P. fragi*, and *Pseudomonas rhizosphaerae*. Similarly to Zhongar Alatau, the *Erwinia* and *Serratia* genera were identified from the *Enterobacteriaceae* family. *Curtobacterium* remained the most mapped genus of the identified *Microbacteriaceae* family, while the number of *Stenotrophomonas* and *Stenotrophomonas geniculata* decreased.

For Ketmen ridge, the majority of the *Enterobacteriaceae* family reads were also mapped to the *Erwinia* and *Serratia* genera, while those of *Pseudomonadaceae* family were mapped solely to *Pseudomonas veronii* and *Pseudomonas viridiflava*. The *Xanthomonadaceae* family returned only *Stenotrophomonas geniculata* and *Stenotrophomonas acidaminiphila* species, and *Curtobacterium* was also present. Distinctive genera included *Ochrobactrum*, *Bacillus*, and *Paenibacillus*.

## 3. Discussion

*M. sieversii*, a wild apple tree originating from Central Asia, is a fruit tree highly valued for its scientific relevance and practical significance. In a study conducted in 2010, it was shown that *M. sieversii* is the main progenitor of current domesticated apple trees [3]. Today, *M. sieversii* is recognized as a key genetic source for improving resilience to environmental stresses, enhancing disease resistance, and contributing unique fruit qualities. Nonetheless, apple trees are negatively impacted by climate change due to global warming, anthropogenic factors, and urbanization. Pathogenic bacteria and other microorganisms contribute significantly to the reduction in wild apple trees.

On the surface and in the internal tissues of apple trees, a diverse spectrum of microorganisms, including fungi, viruses, and bacteria, are found. Given their importance in plant life, including their impact on germination, the immune system, disease resistance, resilience to biotic and abiotic stress, and the general adaptive capacity of the plant, research on the microbiome offers important new perspectives on microbial interactions both among themselves and with the host organism.

Within the framework of the holobiont idea, the relevance of microbiome research is increasingly underlined when considering the presence of bacteria at all levels of the plant—the phyllosphere, endosphere, and rhizosphere. This is especially pertinent for *M. sieversii*, which is the wild progenitor of contemporary grown apple trees, thus providing a fundamental genetic source for knowledge on plant–microbe interactions. Our study of endophytic bacterial communities across three mountain systems in southeastern Kazakhstan revealed clear regional variation shaped by environmental conditions and land use. Zhongar Alatau and Ile Alatau, both located within protected national parks, demonstrated relatively similar and balanced microbial profiles, including taxa with strains known for plant growth-promoting potential. In contrast, Ketmen Ridge harbored a distinct and less diverse community, more strongly dominated by opportunistic and stress-associated taxa.

**Figure 4:**
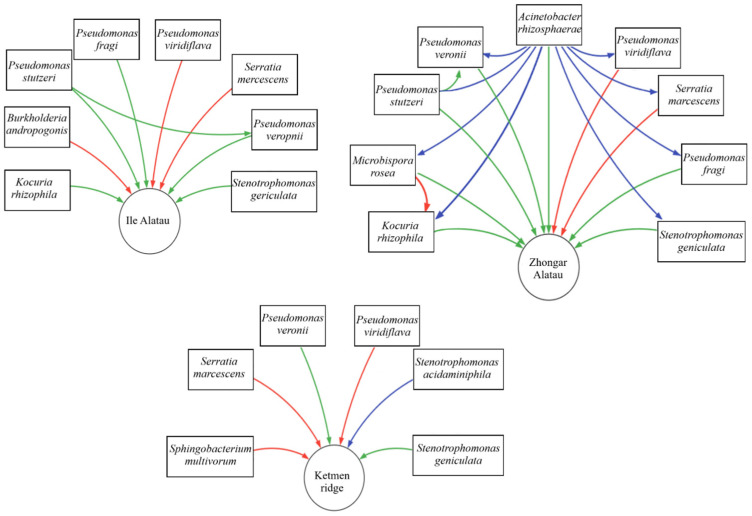
A chart showing bacterial relationships. Red indicates a negative impact, green represents a beneficial impact, and blue signifies a neutral impact or a lack of substantial data.

Several bacterial species detected in the Zhongar Alatau region include species previously described as plant growth-promoting bacteria (PGPB), such as *Kocuria rhizophila*, *Acinetobacter rhizosphaerae*, *Pseudomonas stutzeri*, *Pseudomonas veronii*, and *Stenotrophomonas geniculata*. Numerous studies have reported that specific strains within these species can enhance plant growth and germination by facilitating nitrogen fixation, phosphate solubilization, and other beneficial mechanisms [23]. Some strains are also known to improve plant resilience to environmental stresses by contributing to drought tolerance, increasing antioxidant activity in host plants, and degrading organophosphorus pesticides, thereby supporting phytoremediation efforts [24,25]. The detection of these taxa suggests the presence of a dynamic and adaptive microbial ecosystem that may influence the fitness and ecological success of *M. sieversii* in its native habitat. Notably, *Kocuria rhizophila*, which was also identified in the Zhongar Alatau region (Figure 4), has strains with documented applications in industrial microbiology and bioremediation due to their resilient cell architecture and resistance to chemical pollutants [26].

Notably, *Microbispora rosea*, which was also detected in the region, exhibited an antagonistic effect on *Kocuria rhizophila*, thus suggesting possible microbial rivalry or an inhibitory mechanism that may regulate bacterial population dynamics within the microbial community [27]. Alongside beneficial bacteria, pathogenic bacteria were found as well. *Pseudomonas viridiflava* is a known plant pathogen capable of infecting a wide range of species [28,29,30], including apple trees [31]. It causes necrotic lesions on leaves and stems and root rot but is generally considered an opportunistic epiphytic or endophytic pathogen. *Serratia marcescens* is a globally distributed opportunistic pathogen known to affect plant health [32]. However, some studies classify it as a plant growth-promoting rhizobacterium (PGPR) due to its reported beneficial effects on crops, such as wheat [25,33], lupine [34], chickpea [35], rice [36], and *Arabidopsis thaliana* [37]. Despite these potential benefits, its use in agriculture remains controversial due to concerns about its pathogenicity and potential negative environmental impact.

Though the percentages of the given microorganisms are different, Ile Alatau demonstrated a comparable microbiological landscape. The smallest presence of the *Xanthomonadaceae* family and its main representative, *Stenotrophomonas*, is the most important variation to consider in the bacterial background. Some studies suggested that the members of the *Stenotrophomonas* genus can have a beneficial impact on plants health and development [38], while having an antagonistic relationship with some harmful pathogens [39]. The slightly higher rate of the *Enterobacteriaceae* and *Pseudomonadaceae* family presence could potentially be related to the lower rate of the *Xanthomonadaceae* family presence. The region displayed mostly the same microbial screening results as those of Zhongar Alatau, with the *Enterobacteriaceae* family making up around half of the bacterial volume of the microbiome and the *Pseudomonadaceae* family being the second most abundant representative, accounting for around one-third of the volume. However, in contrast to other regions, Ile Alatau displayed more pathogenic bacteria in its microbiome, such as *Pseudomonas viridiflava*, which is a Gram-negative bacterium that causes stem and flowering issues, thus significantly contributing to the plant’s health decline. The pathogen targets many plant species and leads to the development of bacterial blight. Furthermore, signs of *Burkholderia andropogonis* were also identified, and although the bacteria do not cause economically significant damage, they still contribute to plant health complications [40,41].

The distinct microbial profile of Ketmen Ridge likely reflects harsher ecological conditions, including higher elevations (1734–1845 m a.s.l.), reduced vegetation cover, and exposure to anthropogenic pressures such as grazing. These factors may act as environmental filters, selecting for stress-tolerant and fast-growing bacteria. Although several bacterial species such as *Serratia marcescens*, *Pseudomonas veronii*, *Stenotrophomonas geniculata*, and the opportunistic pathogen *Pseudomonas viridiflava* were found across all regions, Ketmen samples also contained *Sphingobacterium multivorum*, a species previously associated with both pathogenicity—such as causing rot in *Sparassis latifolia* [42]—and plant-beneficial traits, including enhanced drought tolerance in tomatoes [43]. The presence of such dual-role taxa, along with a decline in beneficial families like *Pseudomonadaceae* and *Microbacteriaceae*, suggests a shift in microbial balance likely influenced by environmental stress and interspecies competition. These observations highlight not only the ecological sensitivity of the *M. sieversii* microbiome but also the complex and context-dependent nature of microbial functionality within plant-associated communities.

These findings align with earlier research conducted on the domesticated apple (*Malus domestica*) [44], where endophytic bacterial communities also demonstrated significant diversity, primarily influenced by environmental and genetic factors. Previous research highlighted the functional roles of bacterial endophytes in enhancing host resistance against pathogens such as *Venturia inaequalis*, the causative agent of apple scab, through mechanisms like induced systemic resistance and direct antagonism mediated by bioactive metabolites. In comparison, our current analysis of *M. sieversii* identified numerous beneficial bacterial taxa known to facilitate plant growth and stress resistance, including species such as *Pseudomonas veronii*, *Pseudomonas stutzeri*, and *Stenotrophomonas geniculata*, which include strains reported to confer antifungal properties in *M. domestica*, underscoring their potential role in natural pathogen defense mechanisms [45,46]. However, unlike previous studies focusing predominantly on antagonistic and beneficial bacteria, the present study detected a significant representation of opportunistic pathogens, such as *Pseudomonas viridiflava* and *Serratia marcescens*, highlighting the complex dualistic interactions within the apple tree endosphere. These pathogens may adversely affect *M. sieversii* by compromising tissue integrity, reducing photosynthetic capacity, or inducing stress responses—particularly under harsh environmental conditions. However, co-occurring beneficial taxa may counteract these effects through competitive exclusion, antimicrobial activity, or by priming host defenses [47,48,49]. The co-presence of these microbial groups suggests a dynamic balance that may influence host fitness and ecological success. Further experimental validation is required to determine whether such beneficial taxa can effectively mitigate pathogen activity in *M. sieversii* populations.

For further understanding of the microbiomic interactions and its impact on the wild apple trees population more in-depth research is suggestable. Investigating the fungi community inhabiting the rhizosphere of the trees might unveil more valuable insight into the factors playing crucial role in the plant’s health. The presence of PGPB within wild apple populations suggests potential applications in biotechnological strategies for tree health promote on and restoration ecology. Moreover, the data support the incorporation of microbiome characterization into conservation frameworks, aligning with the holobiont concept to preserve not only the genetic but also the microbial integrity of this culturally and economically significant species. Future studies integrating functional metagenomics and transcriptomic analyses are warranted to unravel the mechanistic roles of key taxa and to explore how microbial dynamics influence the long-term survival of *M. sieversii* under climate change.

It is important to note that the present analysis represents a culture-dependent metagenomic approach. As such, the reported bacterial diversity does not reflect the full microbiome composition of *Malus sieversii*, but rather the subset of microorganisms capable of growing on nutrient agar under the specified incubation parameters.

While this study identified several bacterial taxa with known plant growth-promoting or pathogenic potential, we acknowledge that functional validation experiments were not included. Although the identification of beneficial taxa such as *Pseudomonas veronii*, *Stenotrophomonas geniculata*, and *Kocuria rhizophila* provides a strong foundation, their direct effects on *M. sieversii* seedling growth, nutrient uptake, or stress tolerance under controlled conditions remain to be confirmed. To address this, future studies will prioritize the isolation of dominant strains and evaluate their functional roles through controlled inoculation experiments, phenotypic assessments, metatranscriptomic profiling, and *in planta* assays. These approaches, integrating microbiological, physiological, and molecular markers, will offer deeper insights into host–microbe interactions. Furthermore, expanding the analysis to include rhizosphere fungi may enhance our understanding of the broader microbial influences on plant health. Overall, the detection of plant growth-promoting bacteria in wild apple populations underscores their potential for biotechnological applications in conservation and restoration. These findings support the integration of microbiome research into conservation strategies, consistent with the holobiont concept, which emphasizes the joint preservation of plant genetic resources and their associated microbiota. Functional metagenomics and transcriptomics will be essential to elucidate the mechanisms underlying these interactions and their contributions to the long-term resilience of *M. sieversii* under environmental change.

In addition, while we acknowledge known limitations in taxonomic resolution—particularly for certain bacterial groups such as *Enterobacteriaceae* and *Bacillus*—16S rRNA gene-based analysis remains one of the most widely used and cost-effective methods for profiling microbial communities in environmental and plant-associated studies. Its high-throughput capacity, reproducibility, and accessibility make it especially valuable for comparative and exploratory research. While species-level identification is often challenging due to conserved regions within the 16S gene, several studies have demonstrated that accurate species-level resolution is achievable when using optimized primers, high-quality reference databases, and full-length 16S sequencing [50].

## 4. Materials and Methods

### 4.1. Collection Sites and Sampling

**Figure 5:**
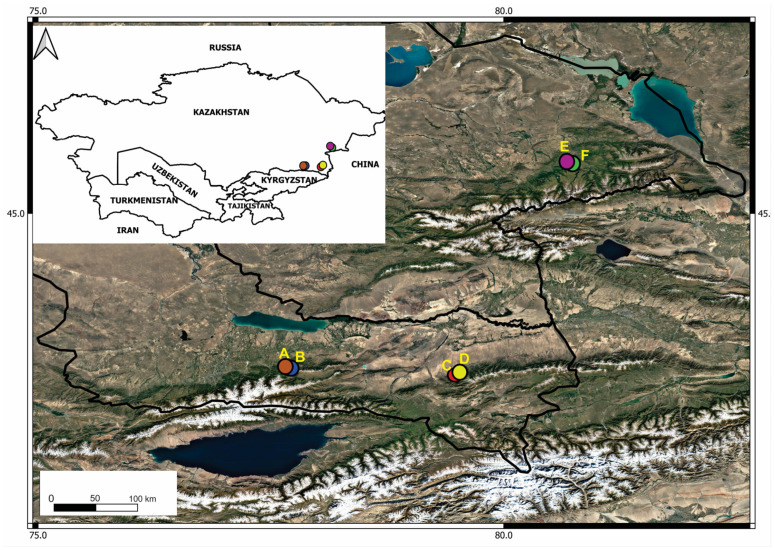
Sampling sites on a map of Kazakhstan. Almaty region: A, B—Ile Alatau populations; C, D—Ketmen populations. Zhetysu region: E, F—Zhongar populations.

Apple tree samples were collected in September 2023, during the ripening season, in 3 different mountain ranges of south-eastern Kazakhstan—Ile Alatau, Zhongar Alatau, and Ketmen (Figure 5). Climatic characteristics of the study regions were assessed based on monthly averages of air temperature and precipitation over the past five years (2020–2024), using data obtained from the official state meteorological database [51]. In the Zhongar Alatau region, the average annual temperature ranges from 8.6 to 9 °C, with the highest values recorded in June–August (21–23 °C) and the lowest in November–January (–1 to –10 °C). Monthly precipitation averages 26–40 mm, with peak values typically occurring in March–April (30–100 mm) and October–November (30–70 mm), while the lowest levels are observed in June (7–30 mm). In the Ile Alatau region, the average annual temperature ranges from 9.5 to 10.5 °C, with monthly maxima in June–August (20–25 °C) and minima in November–January (–1 to –7.9 °C). Precipitation ranges from 36 to 60 mm per month on average, with maxima in March–May (40–110 mm) and October–November (12–80 mm), and minimum values typically occurring in June–September (4–40 mm). In the Ketmen Ridge, the average annual temperature ranges from 8 to 10 °C, with the warmest months being June–August (19–24 °C) and the coldest November–January (–1 to –7.3 °C). Monthly precipitation ranges from 25 to 50 mm, with peaks in March–May (30–110 mm) and October–November (15–80 mm), while June shows the lowest values (4–30 mm).

Sampling in the Ile Alatau and Zhongar Alatau regions was conducted within the boundaries of the Ile-Alatau and Zhongar Alatau State National Parks, respectively, with permission granted by the Forestry and Wildlife Committee of the Ministry of Ecology, Geology, and Natural Resources of the Republic of Kazakhstan. Two populations per region were covered, with samples consisting of branch fragments approximately 10 cm long taken from all four sides of an apple tree, along with 2–3 leaves from randomly selected (simple random sampling) apple trees located at least 5 m apart to preserve spatial variability in microbial composition [52]. Healthy, mature, medium-sized trees were selected. A total of 334 samples were taken with sterile cutting tools and transported in labeled ziplock bags in thermal containers with refrigerant agents at 4–8 °C to prevent the deterioration of plant tissue.

### 4.2. DNA Extraction

The samples of collected branches and leaves were surface-treated under running and sterile water, and the 100 mg samples were then ground in a mortar with the addition of 1 mL of PBS, with 5 replicates per sample. The obtained bacterial suspension was inoculated using the spread plate method onto an NSA nutrient medium (2 g of yeast extract, 5 g of bactopeptone, 5 g of NaCl, 50 g of sucrose, 20 g of agar in 1 L) for 48 h at 37 °C. This study employed a culture-dependent metagenomic approach, which inherently selects for bacteria that can grow under defined culture conditions and therefore does not capture the full microbial diversity. After incubation, colonies were observed, and their morphological characteristics were assessed. Subsequently, the cultured colonies were flushed with 2 mL of TE buffer. A total of 200 μL of flushed suspension was used for genomic DNA isolation.

DNA extraction was performed using an innuPREP Bacteria DNA Kit (Analytik Jena Innuscreen GmbH, Jena, Germany) according to the manufacturer’s protocol minor modifications. The Lysozyme stock solution of 10 mg/mL was prepared beforehand with TE buffer. Approximately 200 µL of bacterial material suspended in TE bufer was used for DNA isolation. Lysis was performed with 15 µL of prepared lysozyme, 25 µL of Proteinase K and Lysis Solution TLS. Following incubation at 70 °C for 10 min, Binding solution was added. Samples were then transferred to a spin filter column, and the bound DNA was washed with Wash Buffers. After the final wash step, residual ethanol was removed by centrifugation, and DNA was eluted in 100 µL of Nuclease-free water. The concentration and purity of the extracted DNA were assessed using a Qubit 4 Fluorometer (Thermo Fisher Scientific, Waltham, MA, USA) with the dsDNA HS Assay Kit. DNA extracts were stored at −20 °C until further downstream analysis, including PCR amplification and sequencing.

The resulting DNA concentration was measured using a Qubit Flex Fluorometer (Thermo Fisher Scientific, USA) with a dsDNA BroadRange Assay kit (Thermo Fisher Scientific, USA) following the required protocol, and DNA was diluted with nuclease-free water to a working concentration of 1–2 ng/μL for PCR amplification.

### 4.3. The Processes of 16S Amplification, Sequencing and Data Processing

Library preparation was conducted using a 16S barcoding kit (16S024, Oxford Nanopore Technologies, Oxford, UK), designed for sequence-based bacterial identification. The samples were combined in sets of 5 per barcode. 16S gene amplification was conducted using manufacturer’s primers provided with barcode sequences. The PCR was conducted on Veriti Pro Thermal cycler 96-well (Thermo Fisher Scientific, USA) for 25 cycles using the program: Initial denaturation—95 °C for 1 min; denaturation—95 °C for 20 s; annealing—55 °C for 30 s; extension—65 °C for 2 min; final extension—65 °C for 5 min. Resulted amplicons were approximately 1500 bp long and assessed by gel electrophoresis with 1.5% agarose TAE (Tris acetate EDTA) gel on a Gel Doc XR+ (Bio-Rad Laboratories, Inc., Hercules, CA, USA). Afterwards, the samples were cleaned using AmPure XP beads, washed with 70% ethanol and eluted in 10 µL of 10 mM Tris-HCl pH 8.0 with 50 mM NaCl. Then, all samples were pooled in a clean 1.5 mL Eppendorf DNA LoBind tube. DNA library quality measurement was carried out using a Nanodrop One spectrophotometer (Thermo Fisher Scientific, USA) and Qubit Flex Fluorometer (Thermo Fisher Scientific, USA) with dsDNA HighSensitive Assay kit (Thermo Fisher Scientific, USA) to adjust the DNA concentration to ~ 5–10 ng/μL. After the addition of the sequencing adapters, the resulting amplicons were sequenced using a MinION Mk1B (Oxford Nanopore Technologies, UK) device, which was managed using MinKNOW software (v24.02.6), with the launch settings of a minimal PHRED score of 9. The generated raw fastq files were basecalled using Dorado (v7.3.9) with a fast basecalling model. Initial quality control was conducted using NanoFilt [53] to extract reads with q > 12 and a length > 280 bp. Taxonomic classification was performed using Kraken2 [54] with the Greengenes [55] database and a confidence threshold of 0.02. Following classification, families with fewer than 200 reads were filtered out. The taxonomic composition was visualized using the “ggplot” and “Pavian” libraries in R (v. 4.4.2).

## 5. Conclusions

This work revealed both common and region-specific microbiome features by highlighting the microbial diversity of *M. sieversii* across Zhongar Alatau, Ile Alatau, and Ketmen ridge. While Zhongar Alatau and Ile Alatau displayed similar microbial compositions, Ketmen ridge showed a clear change with a greater frequency of *Enterobacteriaceae* and a reduction in *Pseudomonadaceae* and *Microbacteriaceae*. This divergence suggests possible microbial antagonism, competitive exclusion, or adaptation to specific environmental parameters, including temperature, humidity, soil pH, and anthropogenic influences. Importantly, the taxonomic profiles included both beneficial plant-associated bacteria. Many beneficial bacteria were identified, including *Kocuria rhizophila*, *Acinetobacter rhizosphaerae*, *Pseudomonas stutzeri*, *Pseudomonas veronii*, and *Stenotrophomonas geniculata*. The aforementioned bacteria help plants develop, increase drought tolerance, and contribute to the bioremediation process. Pathogenic organisms such as *Serratia marcescens* and *Pseudomonas viridiflava* raised concerns regarding plant disease sensitivity. Ile Alatau showed a greater pathogen presence, represented by *Burkholderia andropogonis*, which threatens plant health. Ketmen ridge displayed a distinctive micro-biome picture, which included the bacterium *Sphingobacterium multivorum*, which is connected to both plant development and disease. These results highlight the intricate connections among plants and microorganisms that influence wild apple populations, therefore emphasizing the importance of continually studying microbiome dynamics for agricultural uses and conservation.

## Figures and Tables

**Table 1:** Site coordinates and number of samples.

Region	Site	Coordinates	Altitude (m.a.s.l.)	Number of Samples
Zhongar Alatau	Phenological site	N 45.51963° E 80.73385°	1400	57
	Genetic reserve of Siever’s wild apple trees (Zhng)	N 45.51746° E 80.72224°	1255	52
Ile Alatau	Tau-Turgen	N 43.364244° E 77.680405°	1300	55
	Genetic reserve of Siever’s wild apple trees (Ile)	N 43.36681° E 77.67301°	1585	70
Ketmen	Sumbe	N 43.293056° E 79.484167°	1734	50
	Ketpentau	N 43.305278° E 79.751389°	1845	50

**Table 2:** Sequencing performance statistics.

Mountain Ranges	Number of Processed Reads	Percentage of Classified Reads	Mean Read Length (bp)	Mean Read Quality (PHRED)	N50	Total Bases (Megabases)
Zhongar Alatau	18,549	100%	1426.8	12.2	1461.0	26.5 Mb
Ile Alatau	71,859	99.94%	1327.4	12.2	1441.0	95 Mb
Ketmen	58,325	99.99%	1341.9	12.2	1449.0	78 Mb

## Data Availability

The sequencing data generated in this study are available in the OSF repository at: https://doi.org/10.17605/OSF.IO/QVTRM.

## Supplementary Materials

The following supporting information can be downloaded at: https://www.mdpi.com/article/10.3390/plants14101511/s1, Supplementary Materials contain additional data supporting the findings of this study. Supplementary Table S1 provides detailed information on the sequencing data, including sampling sites, sequencing platform, file names, and links to FASTQ data.

## Abbreviations

The following abbreviations are used in this manuscript:

IUCN	The International Union for Conservation of Nature’s
ONT	Oxford Nanopore Technology
PGPR	Plant growth-promoting rhizobacterium
