# Supplementary material for: Metagenomic Analysis of Wild Apple (Malus sieversii) Trees from Natural Habitats of Kazakhstan
Source: Plants (Basel). 2025 May 18;14(10):1511. doi: 10.3390/plants14101511 (PMC12114784; doi:10.3390/plants14101511)
Supplement: Supplementary file 1 [file plants-14-01511-s001.zip › plants-3617576-supplementary.pdf]

Table S1. Overview of Processed Sequencing Data

№	Region	Site name	Sequencing Platform	Data Link (FASTQ)
1	Zhongar Alatau	Phenological site	MinION Mk1B	https://osf.io/qvtrm/files/osfstorage/67f611f38905d01770cf69f4
		Genetic reserve of Siever's wild apple trees (Zhng)		
2	Ile Alatau	Tau-Turgen		https://osf.io/qvtrm/files/osfstorage/67f612efef4532ea2c8b8024
		Genetic reserve of Siever's wild apple trees (Ile)		
3	Ketmen	Sumbe		https://osf.io/qvtrm/files/osfstorage/67f612b15dcdfb05256ddc26
		Ketpentau		
